# Exploring Aboriginal aged care residents’ cultural and spiritual needs in South Australia

**DOI:** 10.1186/s12913-019-4322-8

**Published:** 2019-07-12

**Authors:** Nina Sivertsen, Ann Harrington, Mohammad Hamiduzzaman

**Affiliations:** 1 College of Nursing and Health Sciences, Flinders University, GPO BOX 2100, Adelaide, South Australia 5001 Australia; 2 College of Nursing and Health Sciences, Flinders University, Adelaide, South Australia Australia; 3 Flinders Rural Health SA, College of Medicine and Public Health, Flinders University, Adelaide, South Australia Australia

**Keywords:** Aboriginal residents, Aged care centers, Healthcare services, Cultural care, Cultural safety

## Abstract

**Background:**

Attention to culture and its impact on health care can improve the quality of care given, add to our understanding of health care among culturally diverse populations, and encourage a more holistic approach to health care within general care. Connection to culture is important to Aboriginal peoples, and integrating Aboriginal culture into general care in residential aged care facilities may contribute to improving care delivery and outcomes for residents. The literature to date revealed a lack of understanding of the capacity of residential aged care and the health practices of carers in relation to providing cultural care for Aboriginal people. This study aimed to explore how cultural care needs are maintained for Aboriginal residents from their own and their carers’ perspectives.

**Methods:**

Applying an Aboriginal centered research method, an Interpretive Descriptive Approach was used as a theoretical framework to explore data in this study. Semi structured audio-recorded interviews were conducted. An additional file provides a complete description of the interview questions used as a guide for the study [see Additional file 1]. Three Residential Aged Care Centres, in South Australia were used i.e., two rural from centres and one urban metropolitan centre. Seven Aboriginal residents and 19 carers participated in interviews. Data was transcribed and an interpretive analysis was employed to code the transcribed data for themes and sub-themes. The study was guided by an Aboriginal community advisory group with an aim to work under the principle of reciprocity; giving back to the communities, participants and those where the research results may have been relevant.

**Results:**

Three themes emerged from the views of the residents and carers: (i) lack of resources and funding; (ii) care practice; and (iii) marginalisation of Aboriginal culture within aged care facilities.

**Conclusion:**

The findings suggest that carers and residents believe cultural inclusion in general care practices may enrich Aboriginal residents’ daily life, health and well-being in residential aged care facilities. This study may provide carers, aged care centre managers and policy makers with information on the need of resources, funding, organised care plan and management, and cultural competency of carers to be considered to improve Aboriginal aged care protocols for integrating cultural care into practice.

**Electronic supplementary material:**

The online version of this article (10.1186/s12913-019-4322-8) contains supplementary material, which is available to authorized users.

## Background

Components of culture have been shown to be integral to the care and well-being of people living in residential aged care [1–3] Cultural care includes issues of history, geography and ethnicity are all important aspects when caring for older people [4]. Cultural care addresses and responds to the needs of an individual experiencing ill health and depression, often because of disconnectedness from place, land, kinship, and spirit or soul [5, 6]. A positive relationship is evident between the cultural care and health and well-being status of an individual [5, 7]. However, cultural care is often absent or neglected in residential aged care centres in Australia [5, 8]. Smith [5] noted a lack of training and competency of carers about both spiritual and cultural care in Australia. Although some researchers have reported on the cultural care needs of older people [5, 9–11], little is known about how these needs of Aboriginal residents’ are maintained in aged care centres.

About 15% of Aboriginal Australians are aged 50 years and over (generally classified as old age for Aboriginal Australians) and less than 1% of people living in aged care centres are identified as Aboriginal [12]. In aged care centres, most Aboriginal people live with chronic diseases, mental health conditions and debilitating co-morbidities [13, 14]. Social and economic exclusion over generations has had extensive and debilitating impacts on Aboriginal health [15, 16]. The health status of Aboriginal people reflects the consequences of history, but healthcare interactions of these people with carers usually begin with an exclusion of an adequate historical background that is important to Aboriginal people [17–19]. This exclusion is exacerbated by an absence of cultural care in the general health care provision at aged care centres. Most Aboriginal people have a strong connection with their family and community members and specific traditional areas of land and rituals [8]. A successful healthcare practice and a culturally safe healthcare interaction requires acknowledgement and acceptance of culture for Aboriginal people by carers [20]. In this study the term carers is used to encompass direct care staff, such as registered nursed, enrolled nurses and carers who provide direct care for residents in residential aged care facilities.

Residential aged care centres in Australia aim to provide responsive and flexible services to Aboriginal people in a way that respects cultural identity and promotes independence, choices and dignity. The new *Single Aged Care Quality Framework – Quality Standards* that replace the Australian Government’s accreditation standards [21], highlights care recipient lifestyle and that ‘Identity, culture and diversity’ are all respected (2018 p.16). This standard is further elaborated to request ‘Respecting a person’s identity, culture and diversity also means: …providing care that is reflective of, and responsive to, their culture…’(2018 p. 16).

Despite these standards, there are problems that Aboriginal people face in accessing cultural care at aged care centres, which are yet to be featured in the literature and in the policies [22, 23]. This study contributes to the literature by providing an insight on the factors and issues that impact on the integration of cultural care into general care for Aboriginal residents.

The average age of Aboriginal people in residential aged care at 30 June 2017 was 73 years, compared with 85 years for non-Aboriginal people [41]. There are approximately 2,672 operational residential care facilities across Australia. Of these, less than 1% (24 facilities) had 50% or more of their clients identifying as Aboriginal or Torres Strait Islander Australians [41]. In general, Aboriginal people access care through various aged care programs. However, some aged care service providers specialise in the provision of culturally appropriate and flexible models of care targeted at older Aboriginal Australians. All residential aged care facilities participating in this study fit under this umbrella.

## Methods

The exploratory research design included an interpretive descriptive approach developed by Sally Thorne [24]. This approach is a non-categorical description, drawing on principles based in nursing epistemological stance, used as a methodology to illustrate the practical and contextualised realities of human health and healthcare needs and practices [24]. Thorne, Kirkham and MacDonald-Emes (1997) considered formal research and interpretation as a foundational fore-structure to a new qualitative inquiry. They stated that a qualitative framework needs to be constructed based on intensive interviews with articulated research participants and critical analysis of the findings in terms of existing knowledge [25]. Given the practice focus of this study, interpretive descriptive approach was found ideal because it would investigate the relationships between the healthcare practices of carers and the capacity of aged care centres [26]. This interpretive description allowed the researcher to frame research questions and prompts and better maintains disciplinary logic and methodological integrity. Following this interpretive descriptive approach, therefore, semi-structured interviews were conducted and data were analysed thematically for this study. An additional file provides a complete description of the interview questions used as a guide for the study [see Additional file 1].

### Research settings and participants

Following community conversations and consultation with the Aboriginal Elders Council of South Australia around appropriate aged care venues, three rural and remote sites, and two urban metropolitan sites in South Australia were chosen. Three of them were willing to participate; two rural centres and one urban metropolitan centre.

The total residents numbered approximately 140 residents, of which around 80 were of Aboriginal descent. Because Aboriginal and Torres Strait Islander people in Australia face multiple health and social disadvantage, they are more likely to develop serious medical conditions earlier in life and have a lower life expectancy than their non-Aboriginal counterparts [41]. In recognition of this demographic, aged care services encompass Aboriginal people aged 50 years and above. The Home Care Packages Program Data Report 2nd Quarter 2017–18 outlines that Aboriginal women in aged care are older than and outnumber Aboriginal men. This outcome appears as Aboriginal women in Australia have a longer life expectancy than Aboriginal men, and so are more likely to need aged care and support.

. Overall, the researchers approached those aged care facilities where residents consisted of Aboriginal people; two facilities had 100% Aboriginal residents, and one facility had approximately 10% of its residents of Aboriginal origin. Two of those facilities had Aboriginal carers/nurses on staff. The research question was to determine for participants in this study, what were the components of cultural care. The results of this study indicated what aspects of culture were important.

Inclusion criteria were residents who (i) identified as Aboriginal; (ii) lived in residential aged care centres; (iii) were able to communicate in English; and (iv) able to provide informed consent. Carers were included if they (i) had experience in providing care to Aboriginal residents; and (ii) were able to communicate in English.

Following ethical approval, the first 26 participants who indicated a willingness to participate and fit the criteria were included in the study. Their ages were not sought but it was accepted that residing in an aged care facility meant they would have been over 50 years of age. The ethnicity of all participants was Aboriginal, and all had resided in the facility for long term care. Overall there were two men and five woman residents, and eight male and 11 female carers. The carers in this facility reflected the demographic of aged care in Australia, and many direct care staff in residential aged care facilities are from culturally and linguistically diverse backgrounds. It is estimated that overall 32% of staff in Australian residential aged care facilities were born overseas and 26% were born in a non-English speaking country [42]. Participants who agreed to be interviewed were assessed at the time of interview that they were able to give informed consent, that their consent was of a voluntary capacity, and that they spoke English sufficiently to undertake an interview.

This research was grounded in an Aboriginal centred research approach using the three principles of (1) enhancement, empowerment and enablement of people; (2) integration and interconnectedness; and (3) control of research, autonomy and self-determination conceptualised by Durie in a Māori context [39]. With a similar history of colonisation and current struggle for social justice, cultural reclamation and the development of Indigenous knowledges, these research principles are equally applicable in an Australian Aboriginal context [40]. The principles were used to inform research processes and practice throughout the research. For example during recruitment this meant allowing space and time for establishing trust and relationships to occur between the lead author, who is Indigenous, and participants. It also meant respect during face to face contact with participants and the encouragement of every participant to question any aspects of the research topic or processes. These measures were instigated to support Aboriginal control of the research and accountability by the researchers, which aligned with the third principle above.

Overall the study was guided by an Aboriginal community advisory group ensuring that the projects’ decision-making processes were directed by awareness and understanding of Aboriginal peoples’ perspectives. It was a priority for this project to focus on how to share the benefits and outcomes of the project with the community - including those who participate in the project and others in the community who may have been affected by the research. Further consideration was given as to how to give back to Aboriginal populations that were central to the research. For the researchers giving back meant that the project members re-visited all sites and communities to share the knowledge generated by this study. The project aimed to be inclusive of Aboriginal perspectives and ensured to incorporate Aboriginal voices as true participants in the project plan.

### Ethical issues

Following ethics approval from the University Ethics Committee and the Aboriginal Health Research Ethics Committee, residents identifying as Aboriginal and carers were approached through contacting managers of each aged care centre. An information sheet was then forwarded to participants along with a consent form. Participants were free to contact the researchers if they wished to be interviewed and were informed that if they chose to withdraw there would be no adverse effects on their care provision, either immediately or in the future. They were also notified that pseudonyms would be used throughout the study to uphold anonymity.

### Data collection

After obtaining consent from seven residents and 19 carers, audio-recorded semi-structured face-to-face interviews were conducted. The interviews occurred in the aged care centres and the length of the interviews ranged from 15 to 45 min.

A semi-structured interview schedule was used to collect data. All interviews began with open ended questions to guide discussion and to allow for the principle of authority and empowered participants by encouraging them to share holistic perspectives of their experiences without being judged. Questions were then followed by prompts for each question. Participants were invited to direct discussion and no conversational points were discouraged or diminished by the researchers. The interviews were conducted by the first and third author and covered topics including standards from Government that direct aged care, experiences of care, persons involved in cultural care, role of carers and support from organisation in meeting cultural care needs.

### Data analysis

In consideration of Thorne’s (2016) view of interpretive analysis of qualitative research, an electronic thematic analysis of both sets of data was conducted. Initially, the audiotapes were transcribed verbatim, and the transcripts were converted to a Microsoft word document subsequently imported into NVivo 11, a qualitative data management software, to facilitate data analysis. Data were analysed following the thematic analysis scheme of cognitive processing – comprehension, synthesise, theorise and re-contextualisation of the data [24]. This analysis of data included four steps: (1) reading and re-reading the transcripts to enhance familiarisation with data and start coding after getting initial insights of codes through automatic code search tools of Nvivo such as auto code wizard, query wizard, word frequency and text search; (2) categorising the codes into sub-themes and themes; (3) defining the themes and sub-themes reflecting on the theory; and (4) review the themes based on the context and extracting the sub-themes and themes. This interpretive analysis of the data provided contextual themes of the findings.

## Results

Three major themes and a range of sub-themes emerged from the seven Aboriginal residents’ and 19 carers’ interviews, which ranged from 15 to 45 min in length. All themes are discussed in the following sections and are: (i) lack of resources and funding; (ii) care practice; and (iii) marginalisation of Aboriginal culture. Each of the themes will be presented with the excerpts that represent the voice of both participant groups of this research.

### Lack of resources and funding

Respondents described the capacity of the aged care centres in relation to the availability of staff, resources and funding. While the number of staff and lack of resources available were identified as factors in views of both Aboriginal residents and carers, the carers indicated that availability of funding is related to the quality of care provision.

#### Staffing challenges

This research confirmed what families and patient advocates have vocalised for a long time; that residential aged care facilities are not adequately staffed. Residents’ and carers’ views in the study were that aged care centres are understaffed, particularly around staff that have adequate cultural training. The understaffing described by the Aboriginal residents and carers related to employee turnover and lack of staff, especially Aboriginal staff members, or staff with specialised knowledge around Aboriginal health and cultures. While one Aboriginal resident defined the employee turnover as the biggest problem, another resident highlighted the low number of Aboriginal staff at the centre taking care of cultural needs:

*They’re not bad but the biggest trouble is with any nursing facility you have a turnover of staff…* (male, resident)

*There is no one (i.e., Aboriginal carer) at the moment …* (male, resident)

Similarly, a shortage of staff in care centres was described by most of the carers. Two carers stated in the following excerpts that the shortage of carers had a negative impact on service delivery:

*… [Place] area only has 1 palliative care nurse. This means that rarely does she/he have time to come for* [place] *terminal clients. The result was that clients who were very sick and at the end of their journey had to be transported to the local hospital for final care.* (male, carer)

*… the care staff, no, there’s no Aboriginal care staff here which is a shame. … As I said there’s not one. Maybe six, seven years ago there used to be two carers here, Indigenous carers, but since then I haven’t seen one at all and again I don’t know why when they’re looking into hiring new carers because we’re getting lots through, why they can’t think oh there’s an Aboriginal carer …* (female, carer)

It was conveyed that the perspectives around lack of adequate staffing was not only about numbers, but also about having sufficient staff with positive and supportive attitudes to Aboriginal culture.

#### A lack of logistic support

Out of 26 nine participants spoke about the availability of resources directly impacting on care in the centres. Most carers were of the perception that there were not enough Aboriginal cultural resources, and that if there had been access to more, then this access would improve the quality of care for Aboriginal residents. The lack of resources was described in relation to; inadequate number of toilets; difficulty in accessing toilets for people in wheelchairs; small room sizes; no schedule for activities or minimal activities offered to residents; distant smoking spaces; problems accessing reflection and relaxation rooms; lack of access to television and relevant Aboriginal television channels such as NITV; lack of table and chairs in outdoor areas around the fire pit; and lack of access to electric wheelchairs. One resident told of difficulties in accessing the toilet and the outdoor smoking area in the following excerpt:

*I used to go there but then I just stopped because…well the one toilet they’ve got there is not handy for people in wheelchairs. When I go to the toilet I have to lean on something … You’ve got your own little room and you’re not in a, where I was last time we were all indoors and it’s like they had a big … where everyone goes in and when people wanted to smoke they go outside or out the back.* (male, resident)

Another resident suggested that access to an electric wheelchair would be helpful in moving around more easily and that this might help her feel more connected to other Aboriginal residents:

*They should give me an electric wheelchair, if they gave me one I’d be happy. An electric wheelchair not one that I’ve got to push around.* (female, resident)

Ensuring mobility and accessible facilities such as specialty rooms may contribute to improving residents’ care experiences within the facilities. A carer commented on the lack of access to facilities:

*… today I had a query from one of the residents that they found it (i.e. relaxation room) locked…* (female, carer)

Thus, both residents and carers were of the view that inadequate logistical support negatively impacted on Aboriginal residents’ connection to culture, and receiving quality care inclusive of cultural care in the aged care facilities were by some seen as compromised and not optimal.

#### Funding

The carers identified a relationship between financial capacity and focus on the Aboriginal culture in care centres. Five carers connected the financial situation of the centres to the lack of integration and celebration of Aboriginal cultural events. Though one care centre was identified by a carer as fully funded, four carers indicated a need for further funding to improve the cultural care for the residents, as one carer stated in the following excerpt:

*It would be absolutely corker if we did get either funding or something where we could be a bit more specific on* [Aboriginal] *culture, not just for the big festivals, but funding to allow* [Aboriginal] *activities on actual day to day activities and what they* [Aboriginal residents] *might need …* (female, carer)

Another carer criticised the role of the government’s commitment and their lack of allocation in State and National budgets for contributing funding towards improvement of living condition for Aboriginal people in Australia:

*If you want to improve something that means you have to be financially able to support* [progress]. *It seems to me the government is a little bit behind … they miss twenty years ago to invest some money to give them* [Aboriginal peoples] *education and [if they had sprung into action twenty years ago then today would] probably have less Aboriginal* [peoples] *who is today on social security, pensions and that sort of stuff, they could be employed and live normal life like everybody else.* (male, carer)

This statement suggests that carers emphasised the importance of action by State and National decision makers, in particular around funding contributing to bringing back Aboriginal culture and Aboriginal cultural activities within aged care centres. This might in turn increase the number of Aboriginal residents and improve their health and well-being. The current capacity of residential care was therefore questioned by the Aboriginal residents and carers in relation to the number of staff practicing within a culturally safe framework, logistic support and available funding to improve cultural foci at today’s aged care centres in South Australia. At present, a perception of lack of funding and lack of integration of cultural care into general care lingers. These experiences were found to contribute to scattered care practices in the views of the residents and carers in the present study.

### Care practice

This theme represented the status of care practice around aspects such as knowledge of care standards, access to care plans, care management and practice of task oriented care. An absence of all-encompassing care practices were evident in the views of the carers and Aboriginal residents, and this made carers and facilities unable to fulfil the current *Single Aged Care Quality Framework – Quality Standard* guidelines that highlight identity, culture and diversity around providing cultural care for the residents.

#### Knowledge about the quality standards of care

Most of the residents did not provide answers or said they did not know about the government directions that focused on the integration of cultural care into general care. Similarly, a majority of the carers confirmed that they did not have sufficient knowledge about the standards of care; they were unsure of what they consist of, implementation of the standards, as well as residents’ rights. Overall, the unfamiliarity with the standards may result in inability to provide holistic care by not enacting the government standards and framework providing cultural care for the residents. The following excerpts were some of the answers when the respondents were asked about their knowledge of the government standards of care framework:

*No, I don’t know.* (female, resident)

*No idea, I’m not the right person to be talking to I don’t think. I can’t give you any information. I don’t know the answers.* (male, carer)

*Whatever the Standard is we abide to it. … Whatever their customs called, whatever, and it doesn’t matter if they are Indigenous we provide here for all cultures.* (male, carer)

However, one resident and some carers demonstrated their knowledge on rights to care by stating that the aged care centre maintained the care standards:

*Yep because if I want anything I tell the nurses and the nurses they know, and I even say to my doctors and they said no it’ll be alright …* (male, resident)

*We are following the Standards and we are trying to help how much we can and they [Aboriginal residents] feel safe and they feel comfortable.* (male, carer)

The lack of knowledge and/or understanding of the government standards made the residents unable to seek appropriate care, and this lack may also disadvantage them from receiving the cultural care they are entitled, in accordance with the standards framework.

#### Care planning

In describing the current care practice, six carers discussed the use of care plans in providing care to Aboriginal residents. Two carers who worked in a multicultural setting stated that components of care plans such as life style, likes and dislikes, personal needs, social history, cultural and spiritual beliefs, and things to do, are important to include when interacting with and providing care to residents. For example, the following excerpts by two carers revealed the nature of care plans they work with on a daily basis:

*We have a care plan so once they do the social history we prepare a care plan for spiritual and cultural* [care] *and every resident’s got* [a plan]*. … That’s why we have the care plans and we get a social history of every client and their cultural and spiritual history from every client.* (female, carer)

*Whenever a new resident comes in we will do a data collection and that’s to know about a person’s history, their lifestyle, what they like, what they don’t like, what kind of activities they would attend and most importantly is their cultural needs and spiritual needs. … I said we have care plans too right, every resident has their own care plans and they would be put in a folder and placed into every* [residents’] *houses …* (male, carer)

Another carer discussed the way care plans guide members of staff to make sure the care is provided according to the needs of residents:

*Whenever I attend that sort of thing I can refer* [to] *their care plan first, the care plan tell me everything* [about] *what they need, what they don’t need, they like, they dislike, everything. Whenever I’m not sure I just check the care plan and I can follow the things what is written in the care plan about their needs and the care they need.* (male, carer)

However, one carer who worked in an aged care centre exclusively for Aboriginal people expressed her frustration about the lack of information of the residents in the care plan. She went on to explain that care plans first and foremost are made by the nursing staff and often only made available to the nursing staff. However she stated that she wished care plans were also made available to carers, as often in this particular aged care setting, the carers were the staff members with most direct contact with residents. Carers thus provided the direct day to day care, whilst nurses had a more managerial role involved only in the most complex care situations including therapeutic regimes involving medications:

*I think we should know more about a resident before they rock up than what we do because I come to work today and there might be a new resident I know nothing about. I’d like to be able to walk in the door and have… say this is a new resident, here’s his or her file, have a browse through and then go from there.* (male, carer)

Another layer of complexity to include culture in care planning was the reluctance to open up or share cultural information. It was found, in particular in situations where non-Aboriginal nurses or carers were involved, that some Aboriginal residents did not want to answer questions related to their culture. One resident described his lack of interest in sharing information about his culture as a result of being tired of repeating information to different people, and because he wanted to avoid having to always explain the different meanings of Aboriginal culture in the society to someone who had no knowledge of Aboriginal culture.

*It’s very hard for me to explain because there’s a lot of meanings out there, and the sacred sites there’s a lot of meanings, there’s a lot of them.* (male, resident)

While the use and regular review of care plans were evident in a multicultural aged care setting, the components of care plans in Aboriginal aged care centres did not adequately cover or incorporate cultural care as expressed by carers participating in this study and illustrated by the resident’s quote above. The lack of culture care plans caused difficulties for carers to consider and include aspects of spiritual and cultural care for residents.

#### Care management

Leadership and communication emerged as two important aspects of care practice in the views of eleven carers. Six carers expressed their satisfaction with the working environment and the leadership relating to care management. In the following excerpt, one carer praised the role of managerial leadership in integrating the Aboriginal culture in care:

*Our boss is very open, has a very open mind about multi-culture so he always makes sure when we organise our activities will make sure all cultures will be included.* (male, carer)

Contrary to this aspect however, was one carer who highlighted that management in their work setting showed an overall lack of understanding about Aboriginal culture due to their cultural differences:

*I couldn’t tell you if the management understood the* [Aboriginal] *culture because obviously the management is from a different culture themselves, pretty much all of them. And I think that that would be part of the reason… is that they barely, probably barely know Australian culture let alone the Indigenous side of it so yeah it’s probably management. … As I said I think the main reasons for that is because the management don’t know what to put into place for them.* (female, carer)

From participants’ views, cultural diversity within the management may have caused a misrecognition of Aboriginal culture, which was also described by the carers as a result of a lack of communication among staff. As previously stated in some settings care plans were created and used by nurses only and not incorporated into carers work. Handovers only included nurses, not carers, in some settings. The views of one carer clarified this lack of communication and how it impacted on the capacity to provide care:

*We don’t have handovers no more, only the nurses. … but we don’t know whether there’s a new resident or who’s here, who’s not, who’s out, who can’t come back in, we don’t get told nothing. I start work at 3 o’clock, I know nothing. I walk in that room, there’s a new bloke in there, I said who are you, like no-one’s told me he’s here, no background knowledge, no nothing.* (male, carer)

Thus, the cultural differences of leadership and a lack of communication among nurses and carers created an isolation of cultural care for some residents in aged care centres. This cultural diversity of leadership also transpired into training and information sessions for staff and carers, ultimately manifested in care practices.

#### Task oriented care practice

According to staff experiences, care practice was frequently related to accommodation, supply of food, and help with showering and general care. This focus on tasks was also conveyed by residents. Two carers emphasized the importance of practical care provided to the residents in the following excerpts:

*They come to us and we do nothing basically, we just allow them for the first time in their life…they’ve got a room to themselves, it’s cool in the summer, it’s warm in the winter and they have clean bed clothes every day, they get clean clothes every day, they’ve never experienced this in their life. They get six lots of food a day; breakfast, morning tea, lunch, afternoon tea, evening meal and supper. Supper is delivered to their room.* (male, carer)

*I think a lot of them if they weren’t here they’d be sitting under a tree somewhere. They get looked after. They get six meals a day and we look after them.* (male, carer)

One resident described her life as routine with not much connection to Aboriginal cultural activities to immerse oneself in on a daily basis:

*Get up, have breakfast and come down to the computer, that’s all, same thing day in, day out. And when they say tea time we all go in there for tea and that’s it, same thing day after day, not much.* (female, resident)

This general task focused care practice was a barrier to integration of cultural care into the general care for Aboriginal residents. The care practice was also unorganised because of staff and residents’ lack of knowledge of the government care standards. The cultural care needs of Aboriginal residents were largely ignored due to lack of information about the residents in the care plans and poor communication processes between leadership and among the carers. Task oriented care practice was prevalent in care centres and resulted in lack of provision of holistic care. Furthermore, cultural unawareness amongst staff contributed to a lack of understanding of the needs of Aboriginal residents.

### Marginalisation of aboriginal culture

The findings revealed a number of issues relating to marginalisation of Aboriginal culture in residential care. These issues included institutional marginalisation, cultural competency of carers and scope of training.

#### Institutional marginalisation

An institutional marginalisation related to a sense that there was not enough emphasis on Aboriginal culture, Aboriginal cultural activities, Aboriginal artefacts or diversity of Aboriginal staff or staff knowledge of diverse Aboriginal cultures in the residential aged care facilities. Two residents expressed their frustration because of a lack of celebration of Aboriginal ceremonies in the atmosphere of multiculturalism and not putting up the Aboriginal flag regularly.

*See even here we have a NAIDOC week which is National Aboriginal and Torres Strait Islander week and they put on a show for us here … That’s the only thing I can think of is NAIDOC week we have … I mean you’ve got Chinese, you’ve got a Japanese woman next door, there’s Yugoslavs, you name it, a lot of Croatians so I don’t think they’d be silly if they started looking after one body, one nation …* (male, resident)

*They should put up the Aboriginal flag more though, I see the Australian flag, I don’t see, like if I’ve got an Australian flag there I want an Aboriginal flag but I can’t get one… so I said pull down them flags, I want an Aboriginal one. More showing the Aboriginal flag around this place you know what I mean.* (male, resident)

One carer confirmed this loss of Aboriginal culture in aged care centres:

*… but like I said that cultural perspective has been lost in our organisation, that’s the part I find difficult. How do we get it back into our facility I don’t have the answers to that. … It’s a bit lost, it’s like a disconnection to the Aboriginal community on the outside, we need to bring it in here to help us understand more and to provide better service for our residents. … We are a nursing home for Aboriginal elders with no culture* [in here]. (male, carer)

In multicultural settings, the emphasis on multiculturalism resulted in a perception that Aboriginal culture was of lesser value, not prioritised sufficiently, and not given enough focus. Multiculturalism may be seen to dilute the importance of showcasing and immersing Aboriginal culture into daily activities and celebrations in aged care. However also in aged care centres with only Aboriginal residents there were experiences of Aboriginal culture being perceived as invisible. This invisibility was related to management and leadership and whether they were seen to be in touch with, connected to, and respectful of Aboriginal culture. If Aboriginal culture was not seen as a priority by leadership, then that belief could possibly lead to encouragement for staff and carers to ignore the cultural care needs of the Aboriginal residents.

#### Cultural competency among carers

According to some carers, cultural competency of staff members, or lack thereof, was often related to language difficulties, lack of knowledge of Aboriginal Australia, but also related to previous professional and personal experiences with Aboriginal culture. Aboriginal residents highlighted that carers with different languages and backgrounds from overseas were more difficult to make a connection. They stated that some carers had difficulties with speaking English. This language barrier was described as a reason for lack of interaction between the carers and the residents. Similarly some carers added to the complexity by explaining that when carers spoke English poorly (i.e. English was their second language) and Aboriginal residents spoke only in their Aboriginal languages this language divide impacted on the quality of care given and received. This excerpt is exemplified in the following comment from a carer:

*… because of her English barrier she can’t communicate, maybe she has a pain and I don’t know what is in the chart for medication she has on daily basis.* (male, carer)

This language difficulty impacted on the residents’ interaction with carers leading to a lack of access to needed care. Some carers also shared from their previous experiences relating interactions with Aboriginal patients or their families. These experiences were described as witnessing disruptive or aggressive behaviours by residents or family members. Such experiences may have influenced carers’ perceptions of Aboriginal people as a whole, as negative or challenging experiences and may have contributed to enforcing stereotypes around drug addictions, aggression or Aboriginal peoples. Education around conflict management and also a reflexive approach to dealing with assumptions, as well as training in how personal worldviews and experiences may impact on our ability to provide culturally safe care is important. The experience the carer had in dealing Aboriginal people influenced their care practice as two carers stated in the following excerpts:

*I experience something when family members come here drunk and asking for too much and sometimes they’re not nice. All of those things, lots of different* [Aboriginal] *people come, under drugs and under alcohol, I think some of them even experience homeless and they come here and spend time making troubles, not big but some issues we have.* (Isaac, carer)

*Yeah when you haven’t seen it* [aggressive behaviour] *before… like yeah to see it yeah but you learn to deal with it. It’s kind of scary but I suppose these things just happen in aged care, anywhere pretty much.* (female, carer)

The perception that carers lack cultural awareness was described by a resident from the perspective that the carers do not prioritise or immerse themselves in Aboriginal residents’ culture due to their personal ambition in relation to career prospects and moving on to other jobs quickly.

*… you don’t get a lot of cultural awareness because the nurses, which is only natural, they want to move onto a higher position or they want to move to do something else in the medical field or whether it’s nursing.* (male, resident)

It was found that language barriers and a lack of cultural knowledge of carers influenced their caring approach to the Aboriginal residents.

#### Lack of scope of education

Most of the carers discussed training and information sessions as an essential part in providing care for the residents. While nine carers highlighted their active participation in training and information sessions, four disagreed that there were sufficient training available and two carers were unsure about the scope of training in the centres. In reply to the question of education provided for staff in the aged care facilities, one carer clearly stated in the following excerpt that there was not enough training and information about Aboriginal culture available:

*No, not me personally and if anyone says that they do* [get offered training] *I’ll be surprised.* (male, carer)

Another carer explained that the nature and components of training were varied and based on choice of the carers rather than focused on the residents’ Aboriginal culture:

*We get sent emails about training and then we individually choose to go to whatever training we wish to go to. And like I said the staff will be getting trained in the Aboriginal culture this year as well so we’ll be a part of that. I think it’s facilitated in what we want, how much of it we want to do and what we want to do.* (male, carer)

Lack of appropriate education available resulted in a limited understanding and capacity of the carers in integrating cultural care when they provided general care. In relation to the marginalisation of Aboriginal culture, there were three issues that emerged in the views of the carers and residents such as institutional marginalisation, a lack of cultural competency among carers and little support from organisations for staff education. These issues resulted in a less than optimum care for the residents.

The theme of marginalisation of Aboriginal culture was related to how, as a result of care received and little Aboriginal context in the aged care centers, residents experienced isolation from other Aboriginal people and communities, and expressed that they felt disconnected from their culture. While institutional marginalisation was evident in settings of multiculturalism, the lack of cultural competency together with a limited scope of cultural training available for carers, contributed to an exclusion of cultural care from general care.

In summary, the views of participants provided three themes that were: a lack of resources and funding of residential care, challenges around care practices and lack of integration of Aboriginal culture into care. The lack of Aboriginal staff, logistic support and scarce funding in residential care impacted on the ability to plan care inclusive of Aboriginal culture. The lack of knowledge around government standards of care, inadequate information in the care plans or limited access to care plans, lack of leadership in regard to promoting Aboriginal culture and communication among carers, and a focus on general tasks in providing care, all resulted in a sense that inclusion of Aboriginal culture was not an important part of care practice. The role of carers and organisations in relation to integration of Aboriginal culture into general care was also defined as exclusionary in terms of lack of institutional policies, as there were no signs of organisational guidelines supporting integration of Aboriginal culture into general care practice. Along with carers needing training around cultural awareness and this training not being readily available, this lack contributed to a sense of absence of cultural care. These factors and issues in combination prevented integration of cultural care into general care for Aboriginal residents.

## Discussion

The findings of the study showed how older Aboriginal residents’ cultural care needs required more attention in some residential care facilities in South Australia. There was a strong connection of Aboriginal people with their culture identified in the literature [27, 28]. The findings provided an insight into how physical and social environment of care centres were of vital importance to accommodate cultural care needs of the residents. The meaning of care to Aboriginal residents was shaped by the physical and social environment of the residential care centres which was composed of the availability of resources and funding for care and care practice by carers. Marginalisation of Aboriginal culture also had an influence on the residents’ access to cultural care. There was a number of limitations in the physical environment of aged care centres according to the views of the participants of this study, including understaffing, a lack of logistics, and scarcity of funding. In Australia, residential aged care centres experience a general shortage of carers and logistics [29, 30]. In this study, the inadequacy in the number of carers was related to employee turnover and a lack of recruitment of Aboriginal staff. Goh et al. (2017) noted that the shortage of Aboriginal carers was the biggest problem in meeting the needs of culturally appropriate care and this finding is consistent with the study’s finding.

In contrast to the literature that suggest there is a lack of aged care specific resources in rural areas of Australia [28, 31], the residents and carers in this study stated that a lack of logistics in both urban metropolitan and rural care centres such as small room sizes, toilets, distant smoking spaces, relaxation rooms, television and access to Aboriginal channels and programs, table and chairs. According to the views of carers, a lack of funding was found to create a challenge for the Aboriginal residents in terms of accessing healthcare and the integration of culture into aged care. This claim is consistent with the findings of other studies that investigated the enhancement of residential aged care’ [31, 32]. The lack of funding, can be assumed as the main reason of a shortage of carers and a lack of logistic support, which play a key role in the relegation of cultural care for Aboriginal residents.

Care management varied between facilities in this study in terms of knowledge about government care standards, care plans, care management and focus of the carers. The findings indicated that both Aboriginal residents and carers were not aware of the care standards accredited by the government, this outcome particularly impacted on the areas of the standards outlining identity, culture and diversity [32]. In essence, little or no knowledge of the care standards resulted in less opportunities for Aboriginal residents to voice their concerns around their cultural care needs and this finding is consistent with other studies that explored Aboriginal people’s healthcare seeking behaviours [33, 34]. When carers were not informed about the standards this information lack impacted on their provision of culturally appropriate care to Aboriginal residents.

In the current context of residential aged care, the findings highlighted issues with care plans around the creation and usage of the plans. The present study also found that the nursing care plans did not contain information about culture of Aboriginal residents or how to incorporate culture into care, and this outcome is similar to this study’s finding [34]. Interestingly this study show a cultural diversity of care managers and lack of communication among carers that resulted in difficulties for leadership to provide integrated care practices and management of routine care. Similar to previous studies, this study also found that the focus of carers was on general tasks (e.g. showers and food) rather than the consideration of specific cultural care [31, 35]. Whilst the knowledge of carers and residents about government standards of care has an influence in seeking and providing cultural care, the care plans lack of inclusion of culture directly led to inadequate cultural care for Aboriginal residents.

According to the views of residents and carers, the Aboriginal culture had been relegated to the margins in some aged care centres in different ways: an institutional invisibility in regards to Aboriginal culture and rituals, lack of cultural awareness and ability to practice and care within a culturally safe framework, and lack of training available for carers. The marginalisation of Aboriginal culture is closely related with lack of funding rather than willingness of the management in aged care centres [2]. However, lack of cultural competency amongst carers was identified as a factor in not providing complete care for Aboriginal residents in this study and in other studies [14, 36].

Adebayo, Durey and Slack-Smith (2017) note how a cultural and linguistic diversity among carers in Western Australia contributed to the absence of culture in general care [37]. One great risk when a cultural divide is an abyss with no bridge on where to meet in the middle, is breakdown in communication. The current study found that carers had experienced that Aboriginal residents had not talked to them, not shared about their Aboriginal cultural background. This breakdown in communication coupled with carers’ lack of knowledge around Aboriginal cultures, contributed to a focus on task oriented care void of cultural inclusion. The fact that some residents chose not to share or talk about their culture with some carers is a sensitive issue, but central to cultural safety. Building trust to foster a relationship was crucial to establishing a partnership between carer and resident. If the residents did not trust the carers to open up conversations, this lack again would lead to a lesser opportunity to provide cultural care. Work needs to be done around how to equip and improve the relationship building between carers and residents, to equip carers who work with Aboriginal patients how to build this trust. With fluctuating staff or understaffing it is difficult to ensure this building of relationships and trust occurring. Training around how to incorporate cultural care is needed [38]. Davy et al. (2017) suggests care models for Aboriginal residents should be developed by upholding Aboriginal people’s identities in connection to spirituality and culture that could build residents’ self-esteem and a feeling of self-worth. But for this outcome to occur, the transactions between residents and carers needs to work based on trust.

Overall the findings of this study are likely to be pertinent to older people residing in aged care, regardless of their ethnicity. However as there has been little guidance in the area of cultural and spiritual care for Aboriginal people from Governments, this study may be useful to policy makers and systems managers. Findings may well be useful in other countries with Aboriginal older populations, particularly countries with similar history and context of colonisation to Australia.

Attention to culture and its impact on health care can improve the quality of care given, add to our understanding of health care among culturally diverse populations, and encourage a more holistic approach to health care within general care. Connection to culture is important to Aboriginal peoples, and integrating Aboriginal culture into general care in residential aged care facilities may contribute to improving care delivery and outcomes for residents.

Given the evidence that cultural care is essential to enhance quality of general care for Aboriginal residents, it is critical for the carers to ensure that cultural care is more consistently evident in service. The findings have several recommendations for general care services including the importance of establishing Aboriginal residents’ cultural care needs and expectations (Table 1).

**Table 1:** Integration of cultural care into general care

Finding	Recommendation
1. Need for change to Aboriginal aged care as the Aboriginal residents showed a strong connection to their identity, families, communities and land.	1. Open ended questions to develop a Care Plan on admission that includes residents’ preferences in regard to: language, name to be used, food choice, sleep pattern, relaxation, prayer times, observing festivals, meeting family members and visiting own places.
2. Carers in this study were not aware of or did not place enough emphasis on the cultural needs of the Aboriginal residents to be included in the care provided.	2. Carers should be educated through on-going information sessions, mentoring, discussions around available literature and critical reflection on their services as culturally safe.
3. The presence of standards of care does not guarantee a culture-centred approach.	3. It is recommended that Management of Aboriginal Aged Care facilities works towards ensuring standards of care are implemented and critique of care philosophy should follow with a plan for making subsequent changes if necessary.
4. This study also highlighted the value of good relationships among and between carers and Aboriginal residents.	4. The employment of more Aboriginal carers and, at the same time, collaborative working among carers in relation to the barriers in integrating cultural care would result in a better and more culturally safe clinical environment.

This study has some limitations: seven residents and 19 carers in South Australia participated in this study and it may not reflect the views of Aboriginal people in other places in Australia. Aboriginal Australia consists of many and varied communities, in rural, remote and urban settings, and as such have different cultural traditions and beliefs. Residents were underrepresented in the participant group, which may be due to age and co-morbidities of the older Aboriginal population. This research did not address the problems in providing cultural care in terms of the centres’ size, location and type of ownership. These limitations may challenge the transferability of the findings, however, interviews with carers from diverse cultural backgrounds and Aboriginal residents at different times and days in different facilities during the interview-period ensured a variety of experiences and perceptions were captured. More studies are warranted to address the influence of an absence of cultural care in general care on Aboriginal residents’ health and well-being. Further whether findings of this study would be similar to other studies that investigates Aboriginal people living in residential aged care centres in other regions of Australia.

## Conclusion

The factors and issues relating to the integration of cultural care into general care were generated from data provided by Aboriginal residents and carers of aged care centers in South Australia. This research provides an insight at organisational and carers’ capacity levels, including a lack of resources and funding, a lack of knowledge around care standards, care plans void of cultural care components, management with diverse focus of care, Aboriginal culture in residential care relegated to the margins, lack of cultural awareness of careers and little scope of training and professional development around Aboriginal culture. These findings provide policy makers and residential aged care center managers with information on how to develop general care protocols to accommodate Aboriginal residents’ culture into care practice, and also informs carers about the needs of cultural competency. However, further research is needed to explore whether the cultural needs of Aboriginal residents are distinctive to South Australia or are common to the Aboriginal residents living in other regions Australia.

## Additional file

Additional file 1: Interview guide. A complete description of the semi-structured interview questions used as a guide for the study. (DOCX 124 kb)

## Data Availability

The datasets used and/or analysed during the current study are available from the corresponding author on reasonable request.

## Abbreviations

NAIDOC: National Aborigines and Islanders Day Observance Committee
NITV: National Indigenous Television
