# Supplementary material for: Exploring Aboriginal aged care residents’ cultural and spiritual needs in South Australia
Source: BMC Health Serv Res. 2019 Jul 12;19:477. doi: 10.1186/s12913-019-4322-8 (PMC6624992; doi:10.1186/s12913-019-4322-8)
Supplement: Supplementary file 1 — Interview guide. A complete description of the semi-structured interview questions used as a guide for the study. (DOCX 124 kb) [file 12913_2019_4322_MOESM1_ESM.docx]

## Additional file 1: Interview guide

There are standards from the Government that direct care within residential aged care services (see excerpt below, read out).

• In your opinion, how does the institution ensure that their care fulfills Standard 3, section 3.8?

• In your opinion, how does the institution enable their nurses and carers to accommodate the cultural and spiritual life of their recipients of care?

• How do the nurses and carers individually make room for culture and spirituality in their care?

Tell me something about what culture and spirituality might mean to you/residents at this time of your/their life journey?

How do the carers/you ensure clients’ needs are met in regard to culture and spirituality?

Have you got an example of how your/clients’ needs in this area were met, that you would like to tell me about?

How does this make you feel?

Is anyone else involved in accommodating culture and spirituality for you/residents, if so who?

What are some of the support you have received from the aged care facility in order to accommodate culture and spirituality for your/residents?

Is there anything that is stopping carers/you from meeting these client needs?

What are some of the kinds of support that you have needed as a staff member (to accommodate clients’ needs of culture and spirituality) since you came to work here?

Is there anything else you would like to add?

*Excerpt Australian Government Accreditation Standards:*

The Australian Government’s accreditation standards (Australian Aged Care Quality Agency 2014, p. 2) highlights care recipient lifestyle and that it is a right to ‘retain their personal, civic, legal and consumer rights, and are assisted to achieve active control of their own lives within the residential care service and in the community’.

This standard is further elaborated on via Accreditation standard 3, section 3.8: ‘Cultural and spiritual life: Individual interests, customs, beliefs and cultural and ethnic backgrounds are valued and fostered'.

According to the National Aboriginal and Torres Strait Islander Flexible Aged Care Program Quality Standards published by the Australian Aged Care Quality Agency (Australian Aged Care Quality Agency 2014) Aboriginal people are entitled to care that meet their needs and respects their dignity and individuality.

According to Government standards, SA aged care facilities aim to provide responsive and flexible services to Aboriginal and Torres Strait Islander Peoples in a way that respects culture, promotes independence, choices, dignity and services.
